# Statistical Analysis of the Performance of Local Veterinary Laboratories in Molecular Detection (rRT-PCR) of Avian Influenza Virus via National Proficiency Testing Performed during 2020–2022

**DOI:** 10.3390/v15040823

**Published:** 2023-03-24

**Authors:** Se-Hee An, Gyeong-Beom Heo, Yong-Myung Kang, Mingeun Sagong, Na-Yeong Kim, Youn-Jeong Lee, Kwang-Nyeong Lee

**Affiliations:** Animal and Plant Quarantine Agency, 177 Hyeoksin 8-ro, Gimcheon-si 39660, Gyeongsangbuk-do, Republic of Korea; ashpri@korea.kr (S.-H.A.);

**Keywords:** proficiency test, avian influenza virus, rRT-PCR, quality assurance, molecular diagnosis

## Abstract

For the early detection of avian influenza virus (AIV), molecular diagnostic methods such as real-time RT-PCR (rRT-PCR) are the first choice in terms of accuracy and speed in many countries. A laboratory’s capability to perform this diagnostic method needs to be measured through external and independent assessment to ensure that the method is validated within the laboratory and in interlaboratory comparison. The Animal and Plant Quarantine Agency of Korea has implemented five rounds of proficiency testing (PT) for rRT-PCR targeting local veterinary service laboratories involved in the AIV national surveillance program from 2020 to 2022. In each round, a portion composed of six or more samples was selected from the entire PT panel consisting of H5, H7, and H9 viruses isolated in Korea and distributed to each participant, and at least one pair of samples was commonly included in each panel for interlaboratory comparison. During the five rounds of PT, a few incorrect and outlying results were detected that required immediate inspection or corrective actions. However, in the quantitative measurement of Ct values, the average standard deviation or coefficient of variation decreased as multiple PT rounds progressed, and a positive correlation between consecutive rounds of PT was observed since 2021. The better consistency or stability in the experimental performance appeared to contribute to the more harmonized results in the latest PTs, and it is assumed that the positive reaction of participants to the challenges of quantitative assessment reports showing their status intuitively might work. We need to continue operating the PT program for local laboratories because they play crucial roles at the front line of the national avian influenza surveillance program, and frequent changes in the human resources or environment for diagnosis in those laboratories are inevitable.

## 1. Introduction

Avian influenza A virus (AIV) is a highly contagious zoonotic pathogen that affects wild waterfowl and poultry. It is divided into high-pathogenicity avian influenza virus (HPAIV) and low-pathogenicity avian influenza virus (LPAIV) based on the disease severity [1]. Wild waterfowl can be infected with all 16 HA and 9 NA subtypes of the virus, while HPAIV is restricted to the H5 and H7 subtypes [2]. The H5 and H7 subtypes of LPAIV can become HPAIV by acquiring mutations during circulation in poultry, which is lethal to poultry and has zoonotic potential through avian-to-human transmission [3,4,5]. Although the symptoms of H9 LPAIV are relatively milder than those of HPAIV, control of LPAIV is also necessary because it can cause economic losses, such as decreased egg production and increased death [6,7]. Moreover, when the stamping out policy is implemented in infected farms and active surveillance needs to be performed in poultry farms in the controlled areas or nationwide in a short time, fast, accurate, and high-throughput detection methods such as real-time RT-PCR (rRT-PCR) are essential. The high accuracy of this molecular detection method is secured by following a valid experimental protocol to prevent false-positive results from contamination and to elude false-negative results caused by rRT-PCR inhibitors, sample degradation, or adopting primers/probes not matching the currently circulating or newly introduced viruses.

In Korea, H5 HPAIV has been continually introduced by migratory birds in the winter season, and H9N2 LPAIV of the Y280 lineage was detected for the first time in the live bird market (LBM) in 2020 [8,9,10,11,12,13,14]. Active and passive surveillance is performed in poultry farms, and in general, the samples are first tested by veterinary service laboratories (VSLs) in upper-level local autonomies. For suspected HPAI cases, the samples are sent to the Animal and Plant Quarantine Agency (APQA) for subtyping and determining pathogenicity. Animal hygiene offices of 17 upper-level local autonomies (9 provinces and 8 special or metropolitan cities) and multiple branches of the animal hygiene office in each province have their own VSLs for the diagnosis of avian influenza. The VSL in the main animal hygiene office of a province works as a confirmatory laboratory for the other VSLs in the branch offices in that province. In each VSL, samples such as oropharyngeal and cloacal swabs, feces, or lysates from the tissues of infected birds are tested for initial detection of the M, H5, or H7 genes with rRT-PCR. All these VSLs and APQA use the standardized ready-to-use M/H5/H7 rRT-PCR kits that are manufactured domestically, and we organize an annual hands-on training program for the new diagnosticians in charge of AIV surveillance in each VSL. However, a national proficiency test (PT) on the diagnostic method, rRT-PCR, is necessary for the harmonization of the diagnostic capabilities of VSLs because there are many VSLs, and the diagnostic results are influenced by many factors including the way of keeping and handling samples, nucleic acid extraction and the setting up of the reactions, as well as the proficiency of the persons.

National or international PT programs have been developed or organized by many reference laboratories domestically or internationally. Depending on the purpose of the PT program or the role of the participating laboratories, the panel of samples may be prepared differently [15,16,17,18]. For instance, the American Public Health Association (APHA) in the US and the Australian Centre for Disease Preparedness (ACDP) in Australia constructed panels to include various influenza strains isolated worldwide and made participants guess the correct answer by using various molecular primers and probes of their own [19]. The APQA has recently participated in the regional proficiency testing provider training, provided by the World Organization of Animal Health (WOAH) and ACDP, to develop and operate the PT program as a reference laboratory of the WOAH in 2022, and the APQA has annually participated in international PT programs for avian influenza conducted by the APHA (Animal and Plant Health Agency, UK) or ACDP since 2019. Domestically, the APQA, as a national reference laboratory, has developed and operated a PT program for Korean VSLs based on the general guidelines for operating a PT scheme of the Regulations and Guidelines of Livestock Disease Diagnosis (notice no. 2019-46, 2019.7.2) or the Commonwealth Scientific and Industrial Research Organization (CSIRO) [19].

In this study, we summarized and analyzed the results of the five consecutive rounds of the PT program that the APQA conducted for all VSLs working together for national AIV surveillance from 2020 to 2022.

## 2. Materials and Methods

### 2.1. Viruses for the PT Panel

The panel viruses for the PT program were selected from the H5, H7, and H9 LPAIVs that were isolated or constructed by the APQA. The panel included two A/Puerto Rico/8/1934(H1N1) (PR8)-derived recombinant viruses, rgBuan2 and rgHD1, which contained the hemagglutinin (HA) genes of HPAIVs of two different clades, A/broiler duck/Korea/Buan2/2014(H5N8) and A/duck/Korea/HD1/2017 (H5N6), respectively [20]. The cleavage sites of these viruses were modified to have monobasic residues consistent with LPAIVs. The panel also included two Korean isolates of different lineages of the H9 subtype, A/chicken/Korea/01310/2001 (H9N2) for the Y439 lineage and A/chicken/Korea/LBM314/2020 (H9N2) for the Y280 lineage. The viruses were propagated in 10-day-old specific pathogen-free embryonated chicken eggs (ECEs) and harvested after 3 days of incubation at 37 °C. The full PT panel consisted of the various avian influenza viruses listed in Table 1, and the allantoic fluid from uninfected ECEs was used as a negative sample of avian influenza.

### 2.2. Preparation, Assessment, and Determination of the PT Panel

The panel viruses were inactivated via heat treatment (56 °C, 30 min), and viral inactivation was confirmed via the twice consecutive inoculation of ECEs. The inactivated viruses were serially diluted 10-fold from 10^−1^ to 10^−5^ with normal allantoic fluids from ECEs, and their Ct values for the M, H5, and H7 genes in rRT-PCR were compared to determine the proper dilution factor for constructing the PT panel. In relation to this, we compared the range of Ct values formed from different combinations of equipment or commercial kits for RNA extraction and rRT-PCR. The appropriate dilution factors for the final panel viruses were decided to be detectable with the different combinations of the equipment and reagents. The final panels were then aliquoted and stored at −80 °C until use.

Specifically, in 2022 for the fifth PT round, three different protocols listed in Table 2 were selected to compare the Ct results according to the different experimental conditions. Two popular models of equipment, Nextractor^®^ NX-48 (Genolution, Seoul, Republic of Korea) and Maxwell^®^ RSC (Promega, Madison, WI, USA), were chosen for panel testing in viral RNA extraction with the NX-48 Viral NA kit (Genolution, Seoul, Republic of Korea Korea) and the Maxwell^®^ RSC simplyRNA Tissue kit (Promega, Madison, WI, USA), respectively. For the ready-to-use rRT-PCR kits for the M, H5, and H7 genes, the VDx^®^ AIV qRT-PCR kit (Median Diagnostics, Seoul, Republic of Korea) and the PowerCheck™ Influenza real-time PCR kit (Kogene biotech, Seoul, Republic of Korea), used by the majority of VSLs, and the two most popular thermocyclers, CFX96 (Biorad, Hercules, CA, USA) and QuantStudio 5 (Applied Biosystem, Waltham, MA, USA), were also employed and tested for the panel viruses.

### 2.3. Homogeneity and Stability Testing of the PT Panel

The homogeneity and rates of deterioration of the aliquoted panel samples were evaluated under shipping conditions in 2022. The homogeneity of the aliquoted samples was tested with randomly selected samples. To assess the stability of the panel samples under shipping conditions, randomly selected panel samples stored at −80 °C were thawed and left at 4 °C for 48 h. The Ct values of the samples were determined at different time points (before freezing, after thawing, and after incubation at 4 °C following thawing for 48 h) to ensure that the difference in the Ct values measured after thawing and after incubation was negligible or endurable during the course of real proficiency testing by the participants. For this, in the three different protocols with the combinations of a thermocycler, reagents, and an RNA extraction method, the results are acquired and compared in Table 2.

### 2.4. Distribution of the PT Panel and Data Collection

The proficiency tests were conducted five times over a three-year period from 2020 to 2022. In 2020 and 2021, two proficiency tests were conducted per year, while a single proficiency test was conducted in 2022. Thirty-five laboratories participated in all of the PT programs, while three other laboratories participated partly. All participating VSLs received a sample panel consisting of six samples, including two commonly distributed samples (CDSs). In 2020 and 2021, split (undiluted and 10-fold diluted) H5 strain samples were used as CDSs, and the composition of the other four samples varied depending on the sample panel, which was randomly allocated to each laboratory. In 2022, however, the sample panels distributed to participants were nearly identical, including the H5 strains used as CDSs. Each laboratory was supposed to submit diagnostic results for its sample panel, including the Ct values for the M, H5, and H7 genes with rRT-PCR, as well as information about the instruments and diagnostic kits used for the test.

### 2.5. Statistical Analysis of PT Results

The diagnostic accuracy of all panel samples was initially evaluated by comparing the submitted results with the assigned values. The quantitative analysis of the Ct values of the CDSs was then compared both within each PT round and among the five rounds. The Grubbs’ test was used to find the presence of outliers in the data, which are defined as values that deviate significantly from the mean of the sample. The test was performed at a significance level of 95% [23,24]. Outliers in the Grubb’s test were identified and excluded from the data in the calculation of the mean, standard deviation, CV (%), and Z-score.

The interlaboratory variability in the diagnostic results was evaluated by calculating the Z-score between the laboratories for the CDSs. The Z-score was calculated using two statistical methods: the classical statistical method that utilizes the average and standard deviation, and the robust statistical method that employs the median and normalized interquartile range (NIQR) [25]. In the first PT round, two laboratories failed to detect the CDSs in the H5 gene assay, and they were excluded from the calculation of the Z-score between laboratories. The magnitude of the |Z-score| was categorized into 3 ranges: less than 2, between 2 and 3, and above 3. A |Z-score| value of less than 2 was considered “acceptable”, a |Z-score| value between 2 and 3 was considered “questionable”, and a |Z-score| value above 3 was considered an “outlier”. Laboratories with questionable or outlier results were subjected to a review of their procedures to identify the reason for the deviation, and corrective action was taken if needed.

The Youden plot was used to visually evaluate the performance of each laboratory by plotting the Ct values of the paired CDSs on a graph with one CDS value on the X-axis and the other on the Y-axis. The Manhattan median, which is the point at which the median of the Ct values of the paired CDSs intersect, was calculated, and a 95% confidence ellipse and 45° reference line was drawn to identify outliers in the Youden plot. The distances of the points from the Manhattan median along the reference line or perpendicular to it were used to estimate the source of error. 

The analysis results of each proficiency test, including the Ct values and Z-scores, were shared with all the participants in a blind manner after each test. After the third PT round, informative graphs were also provided to each group of participants; they were customized at the province or city level. The M and H5 Ct values of a couple of CDSs from all participants were displayed, highlighting the points of the participants who received the graph. This graph provided a visual representation of the results, allowing participants to compare their results with others and identify any deviations or outliers.

To assess the performance among the successive PT rounds comparatively, pairwise scatter plots of the M and H5 Ct values obtained for a CDS between PT rounds were drawn, and histograms, locally smoothed regression, and Pearson correlation coefficients were also generated using the “pairs.panels” function in the psych package (version 2.2.9) in R-4.2.1 software.

The significance of the difference in Ct values was evaluated using one-way analysis of variance (ANOVA) with SPSS statistical software (IBM, Armonk, NY, USA), where a *p*-value of less than 0.05 was considered a significant difference. ANOVA was used to compare the Ct values between different experimental components adopted by each laboratory, such as the diagnostic kit or device.

## 3. Results and Discussion

The APQA conducted five rounds of PT programs between 2020 and 2022 to assess the diagnostic ability of laboratories using rRT-PCR for the M, H5, and H7 genes for national AI surveillance, and most VSLs participated. The panel samples for these PT programs were chosen to reflect the representative strains of the H5, H7, and H9 subtypes isolated in Korea (Table 1) and the laboratories’ abilities to detect avian influenza in a subtype-specific manner were tested. As a component of the panel, wild-type clade 2.3.4.4 H5 HPAIVs were substituted with a recombinant virus, in which the multi-basic amino acid motif in the HA cleavage site was replaced with a mono-basic amino acid for biosafety reasons. All the H9N2 LPAIVs reported in Korea were of the Y439 lineage until 2018, but the Y280 lineage was newly introduced in 2020 and added to the PT panel in the 2021 PT round [14]. Although H7 HPAIV has not been detected in poultry farms in Korea, H7 LPAIVs were also included, since H7 LPAIVs have been frequently isolated from wild birds, and cases of H7 HPAIV in poultry have been reported in Asian countries [22]. Each panel of viruses harvested from ECEs was inactivated and diluted to be used for the PT program. The dilution factor was determined considering the reduction in Ct values during sample freezing and thawing and the range of Ct values produced using the different combinations of instruments and reagents available in the VSLs (Table 2).

When we compared the Ct values with three different diagnostic protocols in 2022, there were no significant differences among the different sample storage conditions for subtypes H5 and H7 (Table 2). Other studies also reported that inactivated panel samples for the PT program were very stable, and there was no significant change in Ct values under different temperature conditions during shipping [15,17]. However, each gene tended to be detected earlier in the thawed samples than in the samples before freezing, and it is presumed that more viral particles were disrupted through the freezing and thawing step, resulting in more genes, and this phenomenon was more noticeable in the Ct value of the M gene in H9 PT samples (Table 2). In the diagnostic protocols, protocol 1 showed significantly delayed Ct values for the M gene compared with the other protocols (Table 2). Between protocols 2 and 3, the Ct values for the M and H7 genes were comparable, but the H5 gene was detected later in protocol 2. This finding suggests that the different protocols employing different extraction methods (protocol 1 vs. protocol 2 and 3) or different thermocyclers and reagents (protocol 2 vs. protocol 3) may affect the analytical sensitivity, and this is also influenced by the states or origins of the samples.

In the first PT round, approximately 30% of the participants used the manual RNA extraction method, but in 2022, the use of automated RNA extraction machines became prevalent, with only one of the participants (two percent) continuing to use the manual protocol for RNA extraction. Regarding thermocyclers, CFX96 (Bio-Rad) or QuantStudio lines (ThermoFisher) were used by most participants, and the ready-to-use rRT-PCR kit made by a commercial company was adopted by approximately 70% of the participants consistently over the 3 years (Table 3).

In the five rounds of PT, only five laboratories submitted incorrect answers at the first result submission. Two laboratories (no. 12 and 17) failed to identify the sample of the H5 subtype in the first PT round, while one laboratory (no. 11) and two laboratories (no. 7 and 37) falsely reported AIV-negative samples as positive in the first and fourth PT rounds, respectively (Table 4). When we assumed that all PT data for CDSs are normally distributed, only one VSL (No. 34) in the fourth PT round was found to be an outlier in the Grubb’s test. For the M and H5 genes of the CDSs, the average Ct values of the VSLs were similar to the Ct values of the APQA across the PT rounds (Table 4). Excluding the outlier from the data, the SD and CV of the Ct values of the CDSs in the fourth PT round were the lowest, followed by the fifth PT round (Table 4). These results may suggest that the overall diagnostic capabilities of the participants are becoming increasingly harmonized as the PT rounds progress.

**Figure 1:**
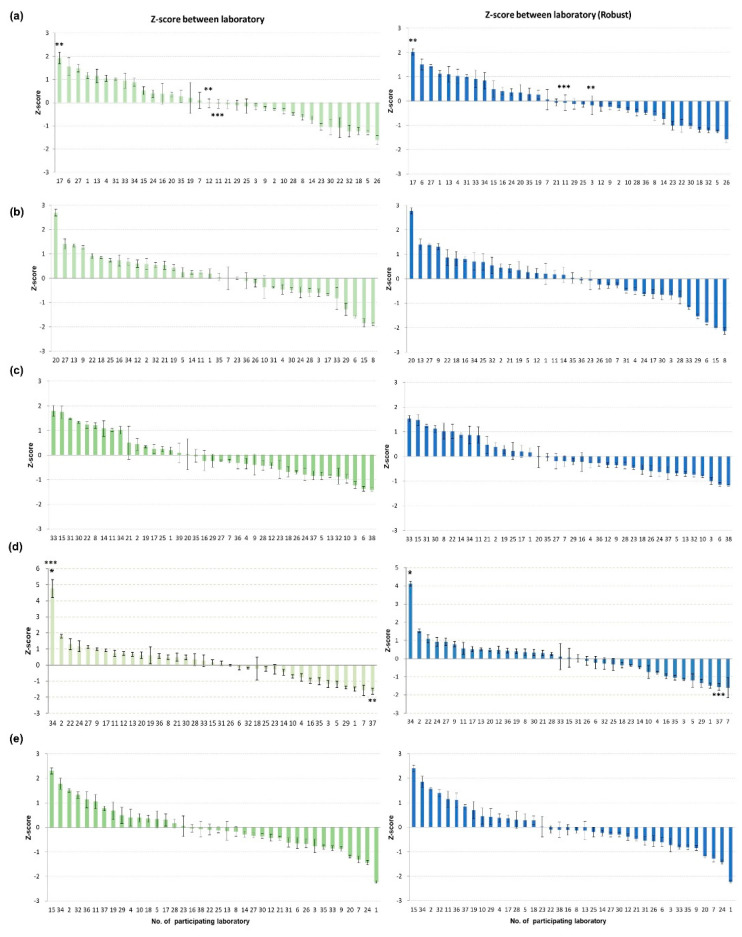
Graph of Z−scores between laboratories during five PT rounds. Z−scores were analyzed for Ct values of the common sample pairs submitted by all participating laboratories in (**a**) 2020 first half, (**b**) 2020 second half, (**c**) 2021 first half, (**d**) 2021 second half, and (**e**) 2022. Z−scores between laboratories were calculated with two methods: classical statistics using average and standard deviation (green), and robust statistics using median and normalized interquartile range (NIQR). * outlier lab, ** lab not detecting H5 gene in CDS, and *** lab reporting false−positive for AI negative sample.

There were VSLs with Z-scores in the upper questionable range: one (no. 20) in the second PT round and one (no. 15) in the fifth PT round and one (no. 8) in the second PT round (with the robust method only). One laboratory (no. 1) in the fifth PT round was in the lower questionable range (Figure 1). Interestingly, the two laboratories (no. 7 and 37) with false-positive results in the fourth PT round had the lowest Z-scores for the CDSs (Figure 1), implying that a higher sensitivity may also increase the risk of obtaining false-positive results.

**Figure 2:**
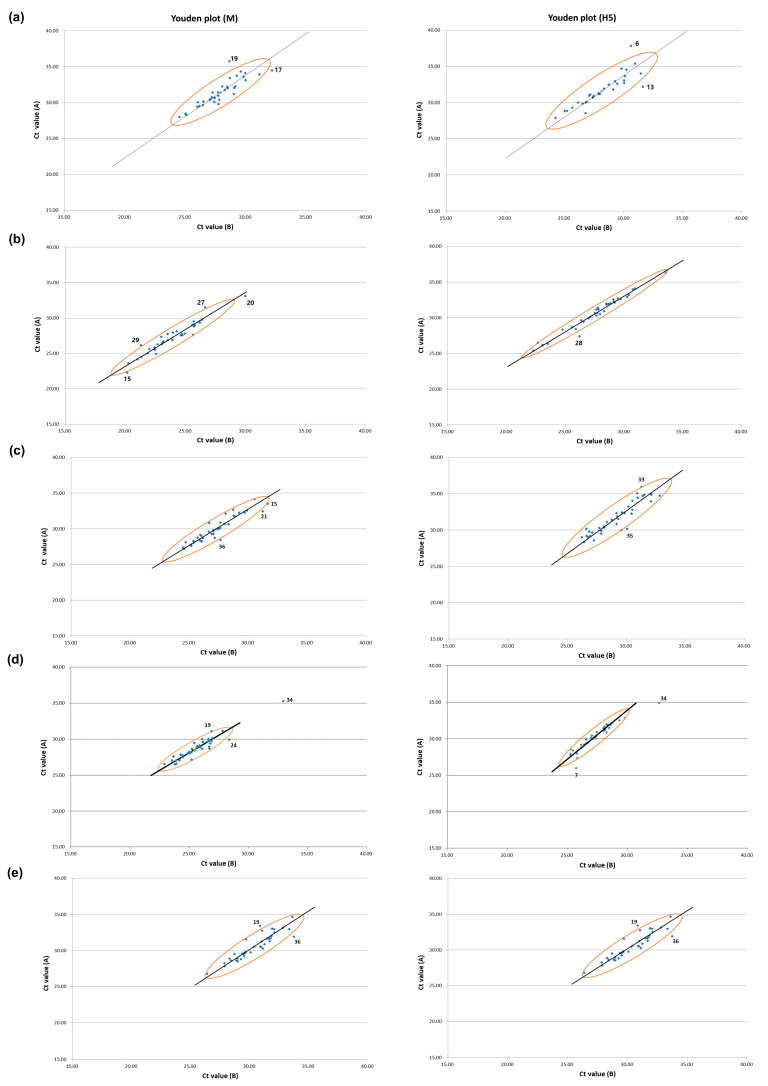
Youden plots of M and H5 genes for laboratories participating in five PT rounds. Ct values for M and H5 genes of the paired CDSs were used to illustrate Youden plot for (**a**) 2020 first half, (**b**) 2020 second half, (**c**) 2021 first half, (**d**) 2021 second half, and (**e**) 2022. The X- and Y-axis values of blue dots indicate the Ct values for target gene of the paired CDSs (Ct value (A) and Ct value (B)). The Youden plots were drawn for M and H5, and 95% confidence ellipses, Manhattan medians, and reference lines were drawn. The laboratories located outside the 95% confidence ellipse were denoted with the number of labs.

For the five rounds of PT, Youden plots, drawn with the Ct values of the paired CDSs for the M and H5 genes, showed that most of the points were dispersed along the reference line (Figure 2). The size of the ellipse area of the 95% confidence level decreased as the PT rounds went on, most noticeably in the fourth PT round. These results suggest that systemic error, or between-laboratory variation, was the main source of error in the PTs, and this bias was greatly decreased in the fourth PT round (Figure 2). Laboratories that were identified as an outlier or in the questionable range in the Z-score analysis were also classified as outliers in the Youden plot, but the Youden plot analysis added more outliers (Figure 1 and Figure 2). This could be primarily due to the moderating effect of using the average of the Ct values of the paired CDSs for performing Z-score analysis in this study, and secondly, the lower confidence level applied in the Youden plot (95%) compared with that for the Z-score analysis (99%). Thus, we could obtain more information about the errors made by each participant from the Youden plot analysis, but this was not used for evaluate the VSLs for formal intervention.

After each round of PT, all participating laboratories received feedback that included their results and those of all other participants. Laboratories whose results were outliers or fell within the questionable range in the Z-score analysis for the CDSs underwent a thorough inspection of their experimental procedures to identify the cause of their results. In such cases, an additional sample panel was provided for retesting if necessary. In particular, laboratory no. 34, which was the sole outlier during all the PT rounds in the Z-score analysis, was known to have left the PT samples at room temperature for several days with negligence, so the VSL was retested with a new panel of samples. After the third PT round, feedback was provided to each participant with a customized and more intuitive graph visualizing all the Ct values for the M and H5 genes of the paired CDSs highlighting the values of the VSLs at the same upper-level local autonomies; the graph was shared with all participants in the same upper-level local autonomies to encourage their coordinated efforts to increase diagnostic capabilities and standardize the instruments or reagents for the harmonization of their data (Figure S1).

**Figure 3:**
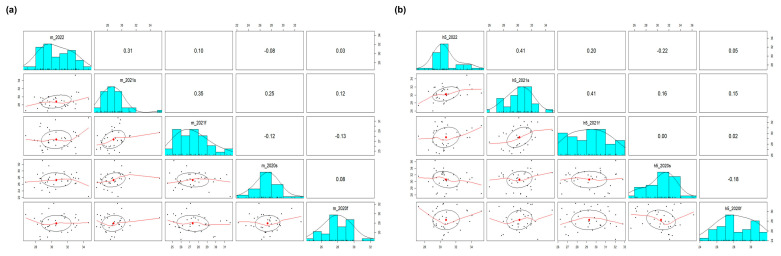
Pairwise correlation analysis of the Ct values of a CDS among the five proficiency tests. Ct values for (**a**) M and (**b**) H5 genes of a CDS from the 36 laboratories participating in all proficiency tests (2020 first half, 2020 second half, 2021 first half, 2021 second half, and 2022) were used to find correlations in their results. The numerical values and red line graphs represent the correlation between the Ct values of labs in two different proficiency tests. The higher the numerical value or the upward−sloping graph, the higher the correlation. The correlation between result of previous PT test and next PT test appeared after 2021 first half, which means that the labs with higher Ct values in 2021 first half showed consistently higher Ct values in 2021 second half and 2022.

The correlation between the Ct values for the M and H5 genes of a CDS was analyzed along the VSLs among the five rounds of PT. The results show a moderate positive correlation between the third and fourth rounds and between the fourth and fifth rounds of PT, with Pearson correlation coefficient values exceeding 0.3 for both the M and H5 genes (Figure 3). This indicates that systemic errors inherent to some laboratories were reproduced while the whole variabilities were decreasing since the third PT round (Table 4). This phenomenon is plausible on the condition that the capabilities of the participant are stabilizing, and we may consider that this is preferable in practice as long as the systemic errors between the laboratories are minimal. This consistency seems to have been generated, in part, as the random error created by manual work decreased. (Table 3). In the multiple linear regression analysis of the H5 Ct values of the CDSs with three explanatory variables (the model of the thermocycler, the method of nucleic acid extraction, and the brand of the diagnostic kit), there were no statistically significant explanatory variables across all the PT rounds supporting the association between the variables and the response, except for the brand of the diagnostic kit in the fourth and fifth rounds of PT (the data are not shown). This could be the reason why the specific brands of diagnostic kits are preferred over others, empirically, by the VSLs, and we assume this signal might stand out in the fourth and fifth PT round because the measurement errors were relatively lower in those last two PT rounds (Table 3 and Table 4).

The data analysis of the PT results indicates a marked improvement in the diagnostic capabilities of the participants in the fourth PT round in 2021. The reductions in SD and CV values and in the sizes of the 95% confidence ellipses of the Ct values of the CDSs in the fourth PT, as well as the positive correlation in the Ct values of the CDSs between the third and the fourth PT, suggest, collectively, that more harmonized and reproducible laboratory test results were produced. We think that the increased uniformity via the use of instruments and more intuitive feedback on the PT results given to the participants might play an important role in this improvement, in which the deviated results and associated variables became more distinguishable. Additionally, it is considered that the revision of the national diagnostic manual for avian influenza, which contained the analysis report of the third PT round results and was reviewed by all the VSLs prior to the fourth PT program, also contributed to these changes. 

However, these favorable changes may be temporary without continued training for novices and without PT programs tightly coupled with informative feedback, as some local officials responsible for AI diagnosis are subject to annual replacement. In the future, it would be necessary to address any association between all the major variables defined in the test protocol and the test results, and this may enable us to provide VSLs with more valuable information and recommendations to enhance the harmonization of the diagnostic results in the national level as a virtuous circle.

## Figures and Tables

**Table 1:** Avian influenza A viruses used for the PT panel.

Strain Name	Subtype	Clade/Lineage	Used Year	Accession No.	Reference
rgBuan2 ^a^	H5N8	2.3.4.4c ^b^	2020, 2021	EPI 509704	[20]
rgHD1 ^a^	H5N6	2.3.4.4b	2020, 2021, 2022	EPI 1123317	
A/Goose/Korea/H277/2022	H5N3	LPAI	2022	EPI_ISL_14161097	
A/duck/Korea/BC10/2007	H7N3	LPAI	2020	EPI_ISL_70556	[21]
A/duck/Korea/H20-2/2020	H7N7	LPAI	2021	EPI_ISL_15505735	
A/wild duck/Korea/H296/2020	H7N9	LPAI	2021	EPI_ISL_3663323	[22]
A/mallard/Korea/H901/2017	H7N7	LPAI	2020, 2022	EPI_ISL_309227	
A/Wild bird feces/Korea/H337/2018	H7N5	LPAI	2022	EPI_ISL_14161096	
A/chicken/Korea/01310/2001	H9N2	Y439	2020	EPI_ISL_13845	[13]
A/chicken/Korea/LBM261/2020	H9N2	Y280	2022	EPI_ISL_492107	[14]
A/chicken/Korea/LBM314/2020	H9N2	Y280	2021	EPI_ISL_492108	[14]

^a^ Recombinant virus with HA and NA genes of H5 HPAIV (A/duck/Korea/HD1/2017(H5N6) and A/broiler duck/Korea/Buan2/2014(H5N8)) with six internal genes of PR8 virus using reverse genetics system. ^b^ Cleavage site was substituted from multi-basic into mono-basic sequence for biosafety in distribution and usage for diagnosis.

**Table 2:** M, H5, and H7 gene-specific real-time RT-PCR results for PT panel viruses in different experimental protocols in 2022 PT round.

Target	Sample		Exp. 1			Exp. 2			Exp. 3		
			Maxwell^®^ RSC simplyRNA Tissue			Nextractor^®^ NX-48 Viral NA Kit VN121			Nextractor^®^ NX-48 Viral NA Kit VN121		
			VDx^®^ AIV qRT-PCR Kit			VDx^®^ AIV qRT-PCR Kit			PowerCheck™ Influenza Real-Time PCR Kit		
			Bio-Rad CFX96			Bio-Rad CFX96			QuantStudio5		
	Subtype	No.	Before Freezing	F and T	4 °C, 48 h	Before Freezing	F and T	4 °C, 48 h	Before Freezing	F and T	4 °C, 48 h
M	H5	1	31.32 ^†^	30.74 ^†^	30.22 ^†^	27.65	26.61	26.58	26.39	25.43	nt ^a^
		2	34.94 ^†^	34.51 ^†^	34.13 ^†^	30.91	29.94	29.99	29.76	27.18	
		3	30.05 ^†^	29.84 ^†^	29.11 ^†^	25.90	24.95	25.50	24.84	25.50	
		4	33.71 ^†^	33.76 ^†^	32.09 ^†^	29.10	28.03	28.48	28.06	29.01	
	H7	5	31.46 ^†^	32.05 ^†^	31.06 ^†^	27.14	26.39	26.65	26.35	27.07	
		6	35.01 ^†^	34.81 ^†^	35.23 ^†^	30.44	29.37	29.60	29.65	30.30	
		7	28.42	26.86	27.60	27.30	25.87	26.21	26.05	26.53	
		8	31.24	30.79	30.70	30.27	29.18	29.87	29.40	29.92	
	H9	9	33.64 ^‡^	29.01	28.68	33.13 ^‡^	31.32	28.96	32.21 ^‡^	29.28	
		10	37.22 ^‡^	32.12	32.16	36.39 ^‡^	31.84	32.91	35.23 ^‡^	32.79	
H5	H5	1	33.28 ^†^	32.56 ^†^	31.95	29.90 ^§^	29.08	29.11	26.89	nt	nt
		2	36.83 ^†^	36.47 ^†^	35.34	33.51 ^§^	32.58	33.66	30.35		
		3	33.70 ^†^	32.29 ^†^	31.53	29.20 ^§^	28.49	29.19	26.13		
		4	37.20 ^†^	35.89 ^†^	34.40	32.68 ^§^	31.56	34.32	29.23		
H7	H7	5	31.99 ^†^	31.95 ^†^	31.22	27.52	27.01	26.80	26.67	26.33	nt
		6	36.02 ^†^	36.05 ^†^	34.07	31.02	30.16	29.90	30.05	29.78	
		7	29.38	28.38	28.60	28.11	27.51	26.67	26.51	25.49	
		8	32.11	31.80	31.85	31.00	30.20	30.03	29.69	28.95	

^†^ Significantly different from the Ct values with the other experimental protocols in the same storage conditions (*p* < 0.05). ^§^ Significantly different from the Ct values with the experimental protocol 3 in the same storage conditions (*p* < 0.05). ^‡^ Significantly different from the Ct values with the different storage conditions within the same experimental protocol (*p* < 0.05). ^a^ nt: not tested.

**Table 3:** Most used experimental instruments and kits in participating laboratories for PT.

Year	Most Common Extraction Method (%)	Most Common rRT-PCR Machine (%)	Most Common rRT-PCR Kit (%)
2020 first half	Manual methods (29%)	Bio-Rad CFX96 (60%)	Kogene biotech PowerChek™ Influenza Virus Real-time PCR kit (72%)
2020 second half	Qiagen Qiacube RNeasy mini kit (33%)	Bio-Rad CFX96 (61%)	Kogene biotech PowerChek™ Influenza Virus Real-time PCR kit (72%)
2021 first half	Genolution Nextractor^®^ NX-48 viral NA kit VN121 (26%)	Bio-Rad CFX96 (58%)	Kogene biotech PowerChek™ Influenza Virus Real-time PCR kit (69%)
2021 second half	Genolution Nextractor^®^ NX-48 viral NA kit VN121 (35%)	Bio-Rad CFX96 (62%)	Kogene biotech PowerChek™ Influenza Virus Real-time PCR kit (76%)
2022	Genolution Nextractor^®^ NX-48 viral NA kit VN121 (50%)	Bio-Rad CFX96 (52%)	Kogene biotech PowerChek™ Influenza Virus Real-time PCR kit (77%)

**Table 4:** Summary of avian influenza virus proficiency test results over 3 years.

Year (Order)	Target Gene	No. of Labs with 100% Correct Answers (%) ^a^	Statistics of a CDS Selected from Every PT Round			
			Ct Value (APQA)	Average Ct Value (Range) ^†^	Standard Deviation ^‡^	Coefficient of Variation (CV, %) ^‡^
2020 first half (1st)	M gene	35/35 (100%)	26.51	27.98 (24.51–32.19)	1.78	5.95
	H5 gene	33/35 (94%)	28.22	28.33 (24.23–31.62) ^c^	2.06^c^	6.76 ^c^
	H7 gene	29/29 (100%)				
	AIV negative	34/35 (97%)				
2020 second half (2nd)	M gene	36/36 (100%)	25.82	27.27 (22.27–33.10)	2.09	8.20
	H5 gene	36/36 (100%)	26.66	27.56 (22.28–33.55)	2.49	8.58
	H7 gene	36/36 (100%)				
	AIV negative	26/26 (100%)				
2021 first half (3rd)	M gene	38/38 (100%)	25.89	27.25 (24.46–31.65)	1.82	6.36
	H5 gene	38/38 (100%)	28.76	29.25 (26.24–32.82)	2.01	6.58
	H7 gene	25/25 (100%)				
	AIV negative	38/38 (100%)				
2021 second half (4th)	M gene	37/37 (100%)	29.35	28.62 (26.49–31.16)	1.23 ^d^	4.58 ^d^
	H5 gene	37/37 (100%)	28.93	27.23 (25.24–29.78)	1.37 ^d^	4. ^d^
	H7 gene	27/27 (100%)				
	AIV negative	35/37 (95 %)				
2022 (5th)	M gene	38/38 (100%)	29.71	30.43 (26.39–33.74)	1.75	5.72
	H5 gene	38/38 (100%)	32.39	30.53 (27.11–34.58)	1.63	5.31
	H7 gene	38/38 (100%)				
	AIV negative	38/38 (100%)				

^a^ Laboratories with incorrect answers for any panel samples were considered to be incorrect. ^†^ Average Ct value and range for one of the commonly distributed samples (original concentration) from all participants. ^‡^ Standard deviation and CV (%) were averages of each result of the commonly distributed samples (CDSs). ^c^ Average Ct, standard deviation, and CV for H5 gene in 2020 first half were calculated excepting the Ct value of laboratories with incorrect answers. ^d^ Average Ct, standard deviation, and CV for H5 gene in 2021 second half were calculated excepting the Ct value of outlier laboratory.

## Data Availability

Not applicable.

## Supplementary Materials

The following supporting information can be downloaded at: https://www.mdpi.com/article/10.3390/v15040823/s1, Figure S1: Scatter plot of the Ct values for CDSs from all participants.

## Abbreviations

AIV	Avian influenza virus
HPAIV	High-pathogenicity avian influenza virus
LPAIV	Low-pathogenicity influenza virus
LBM	Live bird market
VSL	Veterinary service laboratory
rRT-PCR	Real-time RT-PCR
PT	Proficiency test
Ct	Cycle threshold
NIQR	Normalized interquartile range
