# Supplementary material for: Statistical Analysis of the Performance of Local Veterinary Laboratories in Molecular Detection (rRT-PCR) of Avian Influenza Virus via National Proficiency Testing Performed during 2020–2022
Source: Viruses. 2023 Mar 24;15(4):823. doi: 10.3390/v15040823 (PMC10145527; doi:10.3390/v15040823)
Supplement: Supplementary file 1 [file viruses-15-00823-s001.zip › viruses-2273040-supplementary.pdf]

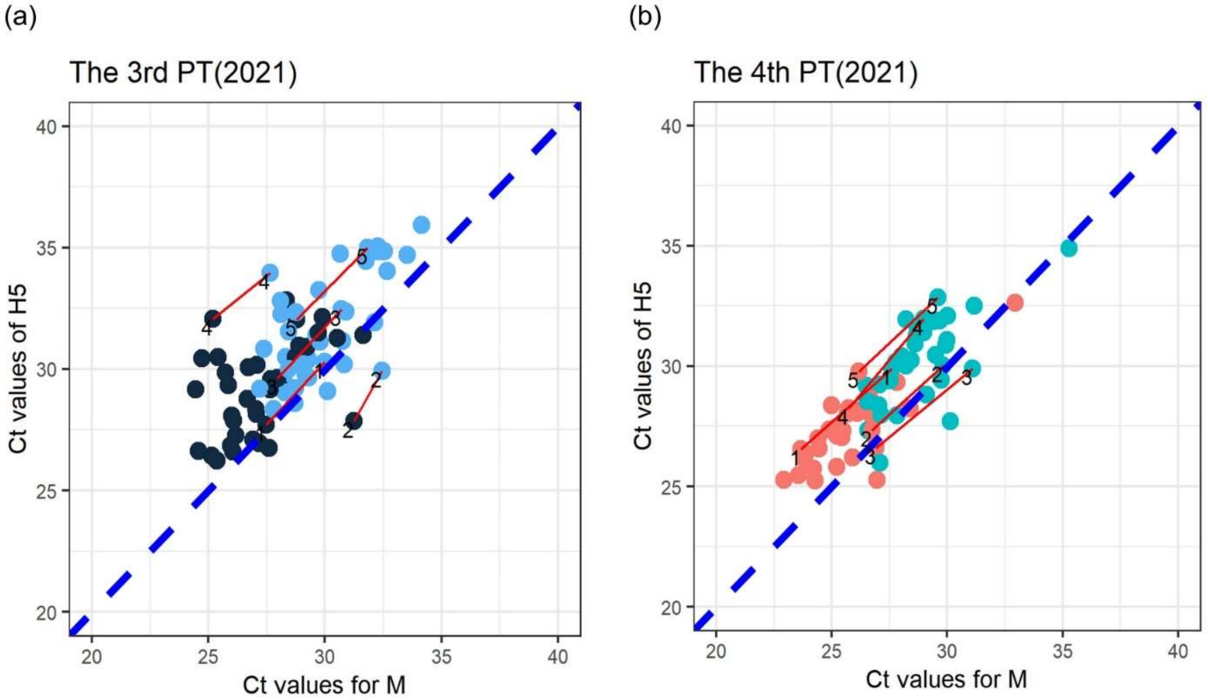

Figure S1. Scatter plot of the Ct values for CDSs from all participants. The Ct values of CDSs, a pair of CDSs with different concentrations but with the same strain, for the M and H5 genes were represented as the X- (M) and Y-axis (H5) values of dots with different colors. The scatter plots for the (**a**) 3rd and (**b**) 4th PT round were given to the 5 participants in the same province as feedback in the post analysis report. The results of 5 labs in same province were converged in 4th PT round after the feedback on the 3rd PT round.
